# Supplementary figures and images for: Social affective behaviors among female rats involve the basolateral amygdala and insular cortex
Source: PLoS One. 2023 Oct 5;18(10):e0281794. doi: 10.1371/journal.pone.0281794 (PMC10553809; doi:10.1371/journal.pone.0281794)

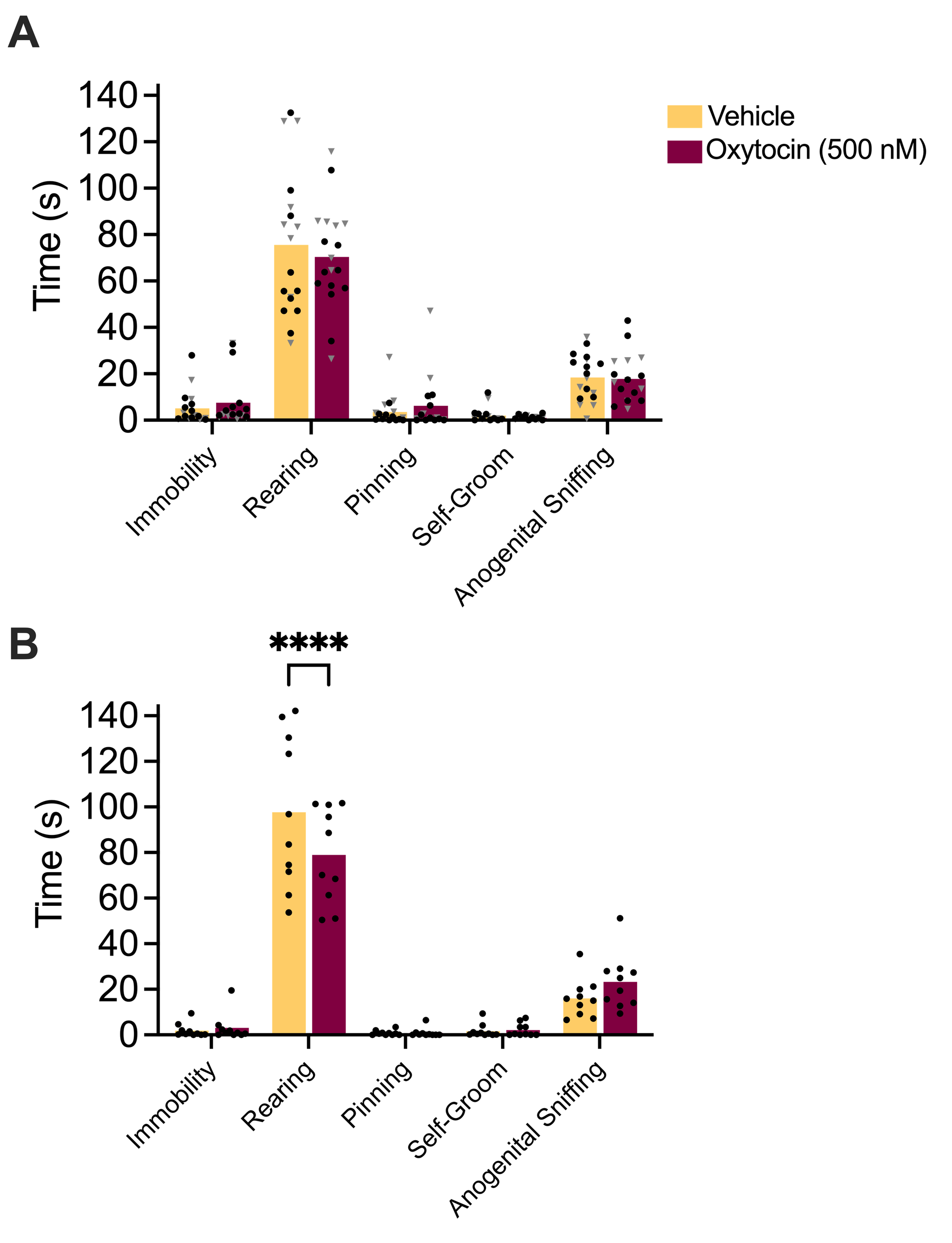

Supplement: S1 Fig — (A) Adult female test rats underwent one-on-one social interaction tests with naive juvenile (n = 10, black circle) or adult (n = 9, gray triangle) conspecifics 15 minutes after infusion of either saline (vehicle) or oxytocin (500nM) in the BLA. 2 Way Analysis of variance (Drug vs. Behavior) revealed no significant main effects of drug or drug by behavior interactions. (B) Adult female test rats underwent one-one-social interaction tests with naive juveniles (n = 10) after infusion of saline or oxytocin to the insular cortex. ANOVA revealed a significant behavior by drug interaction, F(4, 45) = 10.19, p < 0.0001. Post hoc comparisons found that time spent rearing was significantly reduced after oxytocin infusion. (TIF) [file pone.0281794.s001.tif]

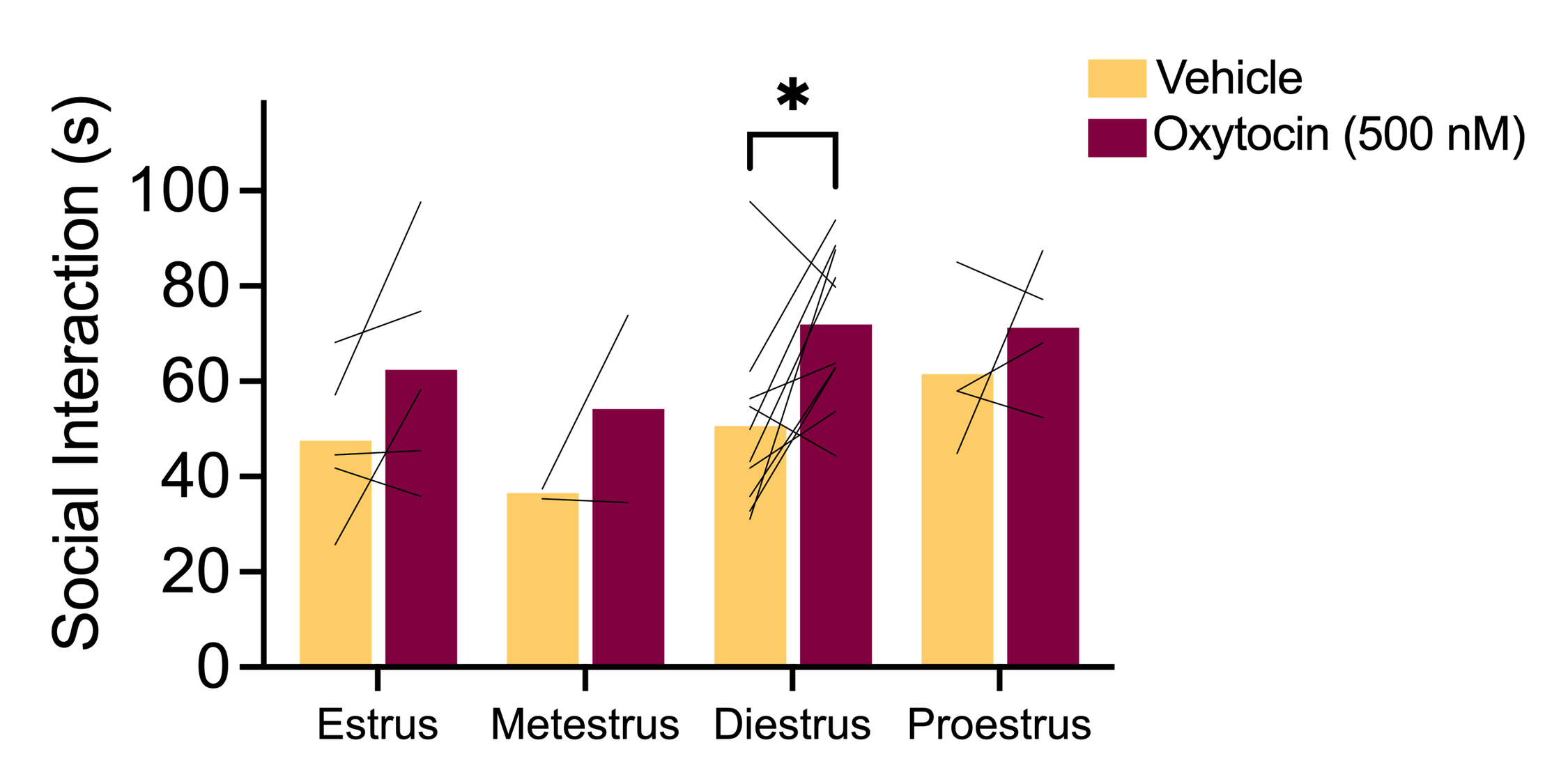

Supplement: S2 Fig — (A) Adult female test rats underwent one-on-one social interaction tests with naive juveniles or adults (n = 21) 15 minutes after infusion of either saline (vehicle) or oxytocin (500nM) in the BLA. All rats received 2 tests on consecutive days and estrus phase was established by vaginal smear after the second social interaction test. Bars indicate mean time spent investigating the conspecific and lines depict individual replicates. A 2 Way Analysis of variance (Drug vs. stage) revealed a main effect of drug (consistent with the analysis in the associated manuscript) and no significant main effects stage or drug by stage interactions. (TIF) [file pone.0281794.s002.tif]
